# Absence labels: How does information about production practices impact consumer demand?

**DOI:** 10.1371/journal.pone.0217934

**Published:** 2019-06-26

**Authors:** Nadia A. Streletskaya, Jura Liaukonyte, Harry M. Kaiser

**Affiliations:** 1 Applied Economics Department, Oregon State University, Corvallis, Oregon, United States of America; 2 Charles H. Dyson School of Applied Economics and Management, Cornell University, Ithaca, New York, United States of America; International Institute of Tropical Agriculture, NIGERIA

## Abstract

Absence labels promote the absence of a particular ingredient or production practice. Consumers usually perceive organic labels as an umbrella absence label for a variety of ingredients and production practices. Such organic labels often use similar language but are based on different certification requirements. For example, both organic wine and wine made with organic grapes are available to U.S. consumers, but little is known about consumer preferences for such labeled products when information about the certification standards is available. Moreover, while absence labels, which advertise the absence of certain attributes or practices, are prevalent on the market, little is known about how information on conventional production practices impacts consumer behavior. Using an artefactual experiment with 128 adult non-student participants, we investigate consumer demand for conventional wine, organic wine, and wine made with organic grapes when information about production standards is provided to participants with and without details regarding conventional winemaking practices. We find that while both organic labels carry a significant and very similar willingness-to-pay (WTP) premium, information about certification standards and conventional wine making practices can reduce WTP for all wines. Providing information about the two organic certification standards reduces consumer WTP for both absence labeled and conventional wine categories. This effect largely disappears for organic wine, but not wine made with organic grapes, when information about conventional wine-making practices is also provided.

## Introduction

Consumers increasingly demand more information about how their food and beverages are produced, which is evident by the rapidly growing availability of voluntary labels on food products [1, 2, 3]. Some of these labels might seem quite similar, making it difficult for consumers to distinguish between them. Some of those labels are absence labels, which promote the absence of a particular ingredient or production practice. Consumers often perceive organic labels as substitutes for GMO-free labels, making organic labels an “umbrella” absence label for a variety of ingredients and production practices [1, 4].

This paper investigates how consumer willingness to pay (WTP) for absence-labeled and conventional products is affected by information about label certification standards, with and without information about conventional production practices. We use an artefactual lab experiment with 128 adult non-student participants to explore how WTP for labeled and unlabeled products is impacted by such information. With wine as a focal category, we find that both organic wines and wines made with organic grapes are associated with significant and similar WTP premiums compared with conventional wines. Providing information about the two organic certification standards reduces consumer WTP for both absence labeled and conventional wine categories. This effect largely disappears for organic wine, but not wine made with organic grapes, when information about conventional wine-making practices is provided along with information about the labeling standards. Overall, consumer WTP for organic and made with organic grapes wines only differs when information about both conventional practices and labeling requirements is available. Our results contribute to the literature on the impact of absence labels on consumer demand in presence and absence of information on conventional production practices.

The USDA organic label and the more generic “made with organic grapes” label reflect substantially different production practices in wine. According to the USDA definition [5], all ingredients must be certified organic and no GM ingredients or added sulfites can be used in organic wine, while in wines made with organic grapes only the grapes need to be grown organically, and sulfites can be used up to 100 parts per million. Conventional wines, on the other hand, can use a GM yeast strain, as well as conventional grapes, caseins, egg whites, and other inputs. Additionally, conventional winemaking allows a higher level of sulfite use.

In most cases retailers do not differentiate between these two organic labels, neither in brick-and-mortar locations, nor online. Wine.com, for example, groups both types of wine under “organic wine,” and provides information about particular certification standards only in the online equivalent of fine print, which consumers can easily avoid. It is likely that the perceived lack of difference between the two labels stems from both a general lack of knowledge about labeling requirements, and the low level of knowledge about winemaking practices in particular. Specifically, consumers seem to know little about additives and ingredients used in wine production [6], which means they might expect grapes to be the only ingredient that could be organic.

Our results yield two main managerial and policy implications. First, our research improves our understanding of whether providing additional production information might change WTP across the board for all wines. This is particularly relevant as the digital disclosure option of the National Bioengineered Food Disclosure Standard allows information provision in addition to the labels. Second, our results inform stakeholders about how consumers value absence labels in the presence of information about conventional production practices. Consequently, these results provide useful marketing information for the wine industry.

## Background and relevant literature

Marketing of the *absence* of particular production processes has become widespread in the food industry [1], but it relies on consumer awareness about particular production practices. For example, some consumers are concerned about genetically engineered (GE) ingredients or antibiotic use in conventional food production [7; 8] and process labels stating a lack of such production practices allow consumers to better identify and avoid consumption of such products. On the other hand, when a label identifies a set of practices that are absent from the production process and a consumer is unfamiliar with the details of that process, the relationship between the label and consumer preferences becomes more complex due to information asymmetry. This necessarily suggests that demand for food and beverages with such “umbrella” process labels cannot be evaluated independently of consumer knowledge about standard practices in conventional product alternatives. Moreover, previous research suggests that, even with individual ingredient or process labels, labeling that indicates the absence of a given characteristic has an asymmetrically lower impact on WTP compared with labeling that indicates the presence of the attribute, likely at least partly due to lack of consumer awareness of the frequency of the use of an ingredient or process in conventional food production [7].

Strong demand for organic foods is well documented in the literature. Hughner et al. [9] review existing research on organic food consumption drivers and conclude that a wide range of motivations, such as health, taste, environmental concerns, food safety, animal welfare, local economic impacts, perceptions of wholesomeness, past traditions, and trendiness all play a role in determining WTP for organic food. WTP premiums have been identified for such products as milk, fruits, and meat (Kanter et al. [10] and Bernard and Bernard [11] both focus on milk; Loureiro and Lotade [12] focus on coffee; Napolitano et al. [13] focus on beef; Van Loo et al. [14] focus on chicken; Krystallis et al. [15] focus on a range of products including olive oil, raisins, bread, oranges etc.). While current research suggests that consumers discriminate between organic labels, those differences seem to be driven mostly by subjective preferences and perceived trust in a given label, rather than objective information or differences in certification standards [14, 16, 17, 18]. At the same time, labels that verify organic and non-GMO status seem to be substitutes for one another [8], as is the case with how other single-process and umbrella labels are perceived by consumers [3].

Food labels often not only inform consumers about the objective characteristics of the production process, but are also perceived signals of quality [19; 20]. As is the case with many process labels, organic labels have been shown to carry “halo effects” for consumers, or, in other words, introduce cognitive bias in consumer decision-making (see [1] for a detailed overview). For example, Vega-Zamora et al. [21] contend that consumers often use organic labels as a heuristic to identify healthier and higher-quality products, without paying much attention to the particular characteristics of any given labeled product.

In general, consumer preferences for wine are complex and driven by a variety of factors. Price remains one of the most important factors in wine selection, and is often perceived as an indication of wine quality. For example, Nerlove [22] using countrywide sales data from the central Swedish wine and alcohol controller, concludes that consumers are highly sensitive to price, holding expert-evaluated quality constant. Lockshin and Rhodus [23] discover a significant disconnect between wholesaler evaluation of Chardonnay quality, based mostly on oak content, and consumer quality evaluation, based only on wine prices. This disconnect prevented wholesalers from accurately predicting consumer WTP for wine. Combris et al. [24] look at wine professionals’ tasting ratings and conclude that while hedonic analysis indicates price is driven by objective characteristics such as vintage year, grape varietal, winery reputation, region, etc., quality evaluations are driven almost solely by sensory characteristics. The sample used in the study included only wine professionals, which is not representative of wine consumers overall, and this disconnect between hedonic and quality valuations is in line with the patterns identified by Lockshin and Rhodus [23]. Veale and Quester [25] use a three by three by three (country of origin by price by acid level) wine-tasting experiment and demonstrate that consumer reliance on extrinsic cues such as price remains extremely robust. In our paper, we remove the influence of individual wine-price/quality cues and instead elicit WTP by inviting participants to bid on wines, thus removing one potential confounding factor in the investigation of WTP and quality perceptions of organic wines.

Research on demand for organic wines and consumer WTP price premiums for wines with various organic labels presents a less consistent picture. Rahman et al. [26] suggest that, despite strong theoretical support for the impact of organic labels on consumer wine-purchasing decisions, wine taste dominates choice and WTP: once consumers tasted wines, sensory characteristics alone determined WTP, with no effect of organic certification. Using contingent valuation methods, Remaud et al. [27] find similar results for most Australian wine consumers, who seem unwilling to pay a premium for the organic attribute. A small proportion of consumers, however, are willing to pay a significant price premium for organic wines in the medium-to-high price category. Remaud et al. [27] provide an interesting snapshot of consumer attitudes towards organic wine, suggesting that at least some consider the organic labels to only be important when applied to food, not wine. The latter point is supported by Mann et al. [28], who find that the organic wine attribute does not appear to be important even for consumers in countries where the organic food share of the overall food market is high.

On the other hand, a contingent valuation study by Poveda et al. [29] suggests that some consumers are willing to pay premiums for organic wines, a result that aligns with the findings of Wiedmann et al. [30], who find that consumers give wine described as organic higher evaluation ratings, even after tasting them. This is also similar to the findings of Waldrop et al. [4], who find positive price premiums in the market prices for organic and various sustainability labels. Conversely, Stolz and Schmid [31] find some evidence that wine consumers expect the taste of organic wines to be inferior to that of non-organic wines, while at the same time expecting organic wines to be healthier. The poor taste stigma associated with organic wine seems to be particularly common in Italy, one of the largest organic wine producers in the world [32]. Furthermore, Van Doorn and Verhoef [33] suggest that organic claims in vice, or indulgent, food categories are associated with lower quality, with the opposite true in virtue product categories. This highlights the possible tension in consumer preferences for wine attributes as well as the conflicting consumer evaluations of attributes of vice and virtue products.

Overall, the evidence pertaining to preferences for organic and conventional wine is mixed. Our paper contributes to the ongoing debate over consumer WTP for conventional wine compared to wine made with organic grapes and organic wine. More generally, our research contributes to literature on the impact of absence labels on consumer WTP in presence of differing certification standards.

### Organic wine certification standards

The USDA recognizes two general organic certification categories for wine: organic wine and wine made with organic grapes. Organic wines are allowed to carry the USDA organic seal or an equivalent foreign seal; wines made with organic grapes can be labeled only with the phrase “made with organic grapes” [34]. The key difference between the two standards lies in regulations governing the use of added sulfites in wine production. Sulfur dioxide, along with some other sulfur compounds, also referred to as generic sulfites, is a chemical compound used to preserve the flavor and freshness of wine by acting as an antioxidant and antimicrobial agent [34, 35]. Sulfites are considered GRAS (Generally Recognized as Safe), and are allowed in food use as preserving agents [36]. According to FDA regulations, sulfite use must be declared when the concentration in food is more than 10 parts per million, and while most ingredients in alcoholic beverages are not declared, sulfite use must be disclosed with the phrase “contains sulfites” [36]. In the U.S., organic wine cannot use any added sulfites, and the overall sulfite level should be below 10 ppm, including sulfites naturally occurring in wine during the fermentation process [35]. Wine made with organic grapes can have a limited volume of added sulfites, up to 100 ppm, while conventional wines in the U.S. can have the maximum of 350 ppm of sulfites [35].

The American sulfite regulations differ significantly from European Union (EU) standards, where organic red wine can contain sulfites up to 100 ppm compared with 150 ppm for conventional red wine and 150 ppm for white wines and rosés compared with 200 ppm for conventional whites and rosés [5, 37]. A wine carrying the EU organic seal might be imported to the U.S. as organic wine (if the sulfite content is below 10ppm) wine made with organic grapes when the sulfite level is below 100 ppm, or conventional wine otherwise ([34]).

This difference in sulfite use across the two certification standards is particularly interesting as consumers are often concerned about sulfite content in wine. A variety of popular press pieces expressing opinions both for and against sulfite use in wine reflect consumer interest in and concern about possible allergic reactions and the health impacts of sulfites in wine. For example, a New York Times article [38] highlights consumer concern about less-than-transparent practices in winemaking that involve additive ingredients, while Kitchn’s article [39] suggests that sulfites are most likely harmless while still pointing readers to sulfite-free alternatives. Wine specialist websites, such as Wine Folly, tend to be firmly on the side of educating consumers about the safety and purpose of sulfur use in wine [40]. On the other hand, some sources, such as *Consumer Reports* [41], suggest that, while no scientific evidence ties sulfite use to wine headaches, they might cause severe allergic reactions and even risk death for a very small portion of the population. Overall, these articles acknowledge consumer concern over the ingredient.

In addition to differences in sulfite levels, organic wines and wines made with organic grapes differ based on certain elements of the winemaking process and the ingredients that must be certified organic. The USDA Organic 101 series installment [34] clarifies that wines bearing the USDA organic designation must use only agricultural ingredients that are certified organic, and non-agricultural ingredients must not exceed 5% of the total product.

Ingredients and processing aids that can be used in winemaking, such as yeast, casein, egg whites, and others, do not need to be organic to be included in conventional wines or wines made with organic grape. The majority of wine consumers are not, however, very familiar with non-grape ingredients used in wine [6]. This might explain why the difference between these two organic certification standards is rarely noted in popular press articles about organic wines, which tend of focus on differences in sulfite levels (e.g. [40]).

Finally, some strains of GM yeast have been approved for conventional winemaking since 2003 [42]. As winemakers are not required to release details about ingredients used in their products, it is hard to estimate the current volume of wine on the market that is produced with such yeast; some specialized wine and organics websites do discuss the use of the ML01 strain in wine production, but general consumer awareness about it seems low.

This paper is the first, to our knowledge, that compares the impact of the two organic certification standards on consumer WTP, with and without information about required standards of production for certifications or details about conventional winemaking practices.

In what follows, we detail the experimental design and estimation approach used in the paper, present our results, and discuss the potential implications of our research as well as directions for future research.

## Methodology and research design

### Research hypotheses

This paper focuses on how absence labels affect consumer demand for conventional and organic wine when consumers are aware of differences in the underlying certification standards for similar-sounding labels, particularly when additional information about conventional winemaking practices is provided. The following hypotheses are developed based on existing research and the theoretical focus of the paper.

*H1*: *Consumer WTP for conventional wine differs from consumer WTP for wine carrying organic labels*.

While we expect consumer WTP for organic wines, wines made with organic grapes and conventional wines to differ, previous literature provides conflicting evidence regarding the direction of the expected differences. While some research suggests that consumers exhibit higher WTP for organic products, including wine [30, 43], other studies indicate that some taste and quality stigmas are associated with organic wines, potentially reducing WTP [31, 44].

*H2*: *Consumer preferences for products with similar but distinct absence labels depend on consumer knowledge about the presence of absence-label ingredients in conventional production practices*.

Previous research suggests that wine consumers, even frequent wine buyers, know very little about winemaking practices [6]. As some organic certification requirements focus on the *absence* of particular winemaking practices (e.g., sulfite use, the absence of non-organic production methods solely for grapes, or traditional grape-growing techniques), information about the potential presence of such ingredients or practices in conventional wine is highly relevant to consumer decision-making.

*H2*.*A As information about conventional winemaking practices is provided*, *the relative valuations of wines with different organic labeling standards will change*.

In particular, as consumers acquire more knowledge about wine ingredients that are not grapes, the distinction between organic wine and wine made with organic grapes will become more significant.

*H3*. *Providing information about production practices will generally decrease WTP for wine*.

Li et al. [45] report a reduction in WTP for wine produced with both conventional and recycled water once any information about irrigation is provided. In general, Lusk and Marette [46] suggest that, when consumer attention is limited, providing information can reduce welfare by increasing search costs and cognitive load. This might impact the utility gained from wine purchases, reducing consumer WTP overall. Additionally, as winemaking and evaluation are often considered as much art as science, reflecting the role of aesthetic experience in the evaluative process [47], detailed information about agricultural production practices might detract from that experience. This is in line with prestigious wineries decoupling a wine’s brand image from internal production practices, and relying to a greater extent on cultural appeals to increase consumer brand appeal while concealing commercial wine production practices [48].

### Experimental design

This study took place in the experimental economics lab at a large northeastern research university, and used the experimental volunteer pool of the lab for recruitment. Cornell University IRB has reviewed the ethical consideration of the research, and ruled the study’s protocol exempt. All participants provided written informed consent for the study participation. One hundred twenty-eight non-student subjects, including general public, faculty, and staff, all over 21 years of age, took part in the experiment and were randomly assigned to one of three groups, with two experimental sessions per group: the control group, with 42 participants; the organic standards information group (referred to as the “organic information treatment” hereafter), with 39 participants; and the organic and conventional production practice information treatment (referred to as the “full information treatment” hereafter), with 47 participants. All participants identified as being at least occasional wine consumers.

Participants were paid $35 for their participation, and could spend any part of this money to bid on six different bottles of wine (three whites and three reds) in computerized non-hypothetical auctions, with zero bids allowable. Red and white bottles of organic wine, wine made with organic grapes, and conventional wine were presented, and organic wines and wines made with organic grapes were clearly labeled as such.

The phrasing used to communicate additional information is provided in Table 1 below. Information for organic certification standards and conventional wine-making practices sourced from USDA [34] and Wine Folly [34, 40, 49], a popular website for wine knowledge, articles authored by the certified sommelier Madeline Puckette. The treatments were designed primarily to study how consumer WTP for labeled and conventional wines depends on consumer differentiation between similar-sounding organic labels, and how preferences for products with absence labels change in the presence of information about conventional winemaking practices.

**Table 1:** Additional information per wine per treatment.

Wine type	Organic information treatment	Full information treatment
Organic wine	Both the growing of grapes and conversion to wine is organic. Other agricultural ingredients, e.g. yeast, are organic. Organic wine is made without genetic engineering. Sulfur dioxide or sulfites cannot be added to organic wine.	Both the growing of grapes and conversion to wine is organic. Other agricultural ingredients, e.g. yeast, are organic. Organic wine is made without genetic engineering. Sulfur dioxide or sulfites cannot be added to organic wine.
Wine made with organic grapes	The growing of the grapes is organic, but not the conversion to wine. Non-organic production methods are allowed for wines made with organic grapes.	The growing of the grapes is organic, but not the conversion to wine. Non-organic production methods are allowed for wines made with organic grapes.
Conventional wines	No information	Conventional winemaking allows for the use of non-organically produced grapes and other agricultural ingredients, such as yeast, casein, egg whites, and others. GM yeast (ML01) has been approved for use in the U.S. since 2003. Sulfur dioxide or sulphites can be used in conventional wine.

While additional information differed by treatment, all participants were shown the bottles of wine at the front of the room as well as a photo of each wine bottle during the bidding process on their individual computer screens. The bottles were not modified in any way from how they look in a real retail environment. Each wine label included the name of the wine, the year of the vintage, country of origin and varietal information. The same six wines were used in the three treatment groups—in the control group, no information beyond that was provided. All wines used in the experiment came from small boutique wineries that were less likely to be familiar to participants before the experiment.

Each session of the experiment started with an explanation of the experimental procedures, including details regarding participants’ ability to withdraw from the study at any time, the consent process, Internal Review Board (IRB) approval and contact information, and the general functioning of the economic experiments. The Becker, De Groot, Marschak mechanism (BDM [50]) was used as the auction elicitation method to limit competitive bidding among participants. Using this mechanism, participants submitted their WTP for an item in a sealed bid though their computers. Next, a randomly drawn price (one per session) for the product was drawn from a predefined price distribution, and if the drawn price was below or equal to the sealed bid, the participant received the item at the drawn price. If the drawn price was above the submitted maximum WTP, the participant did not purchase the item. Typically, with the BDM-style auctions, participants are aware of an approximate market price of an item that is being auctioned. Wine prices, on the other hand, cover a wide range of potential values. Because of this, in all three treatments, participants were told that the market price of wines used in the experiment ranged between $15 and $35, but no details on actual individual prices or relative prices of specific wine bottles were provided. This enabled us to reveal some information about a general tier of wine quality without providing individual price/quality cues for any of the wines used in the experiment.

A $1 bill was used in the practice round to demonstrate why it is in subjects’ best interest to submit their true maximum WTP in the auction. In the case of a $1 bill, the optimal bid in such an auction is $1.

After the practice round, subjects proceeded to place their bids on the six wines used in the experiment. One random auction out of six wine auctions was implemented; participants were advised that each of them could leave with at most only one bottle of wine. The random implementation mechanism is generally considered to be incentive compatible under monotonicity assumptions [51].

A detailed computer-based socio-demographic survey followed the auction element of the study. In addition to more standard socio-demographic questions on gender, age, household income, etc., the survey also included questions about wine consumption habits and organic consumption and shopping preferences.

### Descriptive statistics

Table 2 provides detailed summary statistics indicating the socio-demographic characteristics of the participants. As mentioned above, this information was collected via a brief survey following the auctions for the wines. In general, the majority of our sample participants were female, Caucasian, and consumed wine less than once a week. Approximately 40% of the participants reported having children, 44% had completed a college degree, and 47% reported household incomes of between $40,000 and $79,999. The average age of the respondents was 37 years, with 10% reporting that they never buy organic foods, 5% reporting that they buy only organic foods, and around 8% reporting sulfite intolerance. Most of the participants (86%) reported that they pay attention to food and beverage labels, and around 30% reported having attended some type of course in food science or nutrition.

**Table 2:** Descriptive statistics of demographic variables by treatment.

	All	Control	Organic Information	Full Information
WTP	11.78	13.24	10.16	11.82
	(5.59)	(5.55)	(5.15)	(5.63)
Age	37.461	36.00	35.18	40.660
	(13.59)	(12.016)	(12.983)	(14.805)
Female (%)	78.12	69.05	84.62	80.85
Have children (%)	39.84	28.57	43.59	46.81
Caucasian (%)	68.75	59.52	76.92	70.21
African American (%)	5.47	4.76	7.69	4.26
Asian (%)	17.19	28.57	12.82	10.64
College degree (%)	43.75	45.24	41.03	44.68
Master’s degree (%)	17.97	9.52	20.51	23.40
Income less $40,000 (%)	13.28	14.89	5.13	14.89
Income $40,000-$79,999 (%)	46.88	47.62	53.85	40.43
Never buy organic food (%)	10.16	12.82	10.64	10.16
Always buy organic foods (%)	5.47	4.76	5.13	6.38
Taken a food science or nutrition course (%)	30.47	35.71	28.21	27.66
Usually read labels (%)	86.72	92.86	84.62	82.98
Drink wine less than once a week (%)	73.44	76.19	76.92	68.09
Drink wine 2–3 times a week (%)	21.09	14.29	23.08	25.53
Have a sulfite intolerance (%)	7.81	11.90	10.26	2.13
# of bids	768	252	234	282
# of bids≤$5.00 (10^th^ percentile)	98	21	43	34
# of subjects	128	42	39	47

While assignment to the treatment groups was random, some imbalance in observed demographic characteristics was present across treatment groups. For example, the control group had significantly fewer females, participants with children, a master’s degree, or drank wine 2–3 times a week. Participants in the full information treatment tended to be slightly older, and were less likely to have reported sulfite intolerance. Finally, fewer participants in the organic information treatment had household incomes below $40,000 compared with participants in the other groups. While those characteristics are not correlated with assignment to treatment, we addressed the imbalances by including model specifications that control for observable demographic characteristics, which we discuss in greater detail in the next section.

## Results

### Participant bidding behavior

**Fig 1:**
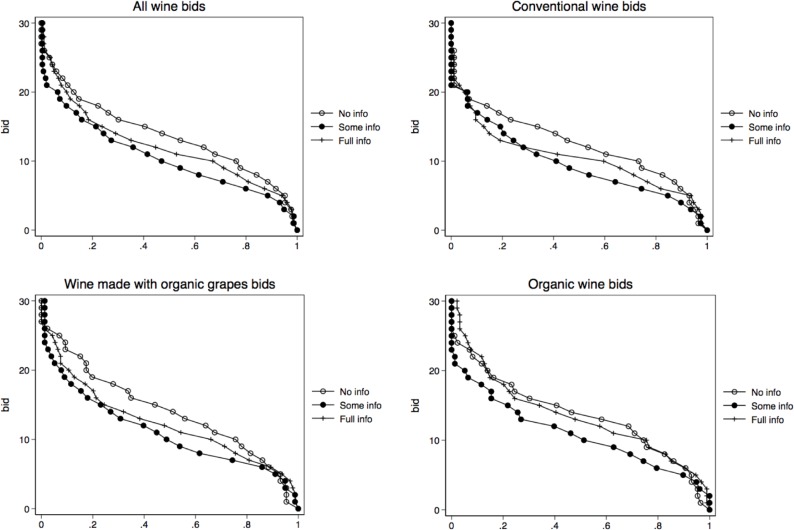
Cumulative demand by treatment. Note: X axis represents cumulative proportion of bids equal to or below any given dollar amount. Bids are reflected on Y axis.

While the hypotheses formulated in the previous section are tested using econometric estimation approaches detailed under “Model Specification” below, we first examine participants’ bidding behavior by graphing demand curves for conventional wine, wine made with organic grapes, and organic wine in all three treatments (see Fig 1).

The first look at the demand graphs suggests that the demand for conventional wine indeed is lower than the demand for organic wine or wine made with organic grapes. Additionally, the graphs suggest that any additional information reduces, to varying degrees, WTP for conventional wines and wine made with organic grapes, but not for organic wine.

We check whether the differences evident in the graphs are statistically significant, and find support for this. Average bids for organic wine are not statistically different in the full information treatment and the no information treatment (p = 0.7729). On the other hand, WTP for conventional wines and wine made with organic grapes is significantly lower when any information is provided (p<0.0000).

Cumulative demand graphs also suggest that very few participants in any treatment were willing to pay more than $20 for any wine. However, bids over $20 are more common for organic wine and wine made with organic grapes than for other wines. This can be seen by comparing the proportion of bids over $20 on the conventional wines panel of the graph to the proportion of bids over $20 in the organic and made with organic grapes graph panels. The X axes on the graphs reflect proportions of bids equal to or below any given price, while the Y axes indicate bids in dollars.

### Model specification

Since the participants in our experiment were allowed to submit zero bids, we estimate a Tobit regression model for left-censored dependent variables. This is a standard approach to accommodate a truncated distribution of bids [52, 53]. Similar specifications were run with an Ordinary Least Squares regression with clustered errors, and the results parallel the Tobit results presented herein and the main results and the conclusions of the paper stand.

We estimate the following Tobit model to identify the impacts of treatment, label, and their interaction on consumer WTP for wines. It is worth noting that the estimated coefficients for dummy variables in the Tobit model can be directly interpreted as the marginal change in WTP compared to the control case, when the dummy variable is equal to zero.

$$\left\{ \begin{array}{c} {WTP}_{ijt}^{*}=\alpha_{it}+\beta_{red}Red+\beta_{j}{Label}_{j}+\beta_{t}{Treatment\;Dummy}_{t}+\\ +\beta_{jt}Labelx{Treatment\;Interaction}_{jt}+\sum_{l}\gamma_{l}x_{li}+\varepsilon_{ijt}\\ {WTP}_{ijt}=\max\left({WTP}_{ijt}^{*},0\right) \end{array}\right.$$

Here, subscript *i* refers to a subject, *j* to a certification standard (the omitted standard is conventional, with two dummy variables for organic and organic grape wines), and *t* represents the experimental treatment. The constant is denoted as *α*_*it*_, *Treatment Dummy*_*t*_ is the treatment identifier, and *Label*_*j*_ denotes whether the bid was for wine made with organic grapes, or organic wine. *β*_*j*_ and *β*_*t*_ are estimates of treatment and label impacts on consumer WTP, while *β*_*jt*_ captures the differential impacts of treatment on organic wines and wines made from organic grapes. *γ*_*l*_ represents the marginal effects of socio-demographic attribute *l* on WTP, while *x*_*li*_ is the demographic attribute *l* for individual *i*; finally, the error term is *ε*_*ijt*_~*N*(0,1). We ran multiple specifications of the above model that included a different set of control variables and obtained estimates that were consistent and robust to different specifications. While, as expected, the fit improves as we control for more respondent specific characteristics, the first specification only includes fixed effects for red wine, and its results are consistent with the model controlling for socio-economic background of respondent, wine consumption habits and organic shopping habits, both in sign and in magnitude. This suggests that our results are robust to multiple alternative specifications.

Another way of looking at changes in consumer WTP due to information about label certification standards and conventional production practices involves examining very low bids on wine. All participants knew that the general price range of the wines used in the experiment was between $15 and $35. However, some consumers submitted bids that were significantly below $15. While it is common in experimental auctions to see some underbidding, as we might see bids from participants who are not target consumers of a particular item on the market, we want to identify patterns of low bids across treatments.

$${LowBid}_{ijt}=\left\{ \begin{array}{c} 1\;if\;{WTP}_{ijt}^{*}<5\\ 0\;otherwise \end{array}\right.$$

To do that, we distinguish between the bottom 10^th^ percentile of bids, which are bids at or below $5, or *LowBid*_*ijt*_ = 1 and the rest of the bids and use a Probit model to investigate the most important determinants of the probability of submitting a low bid. The dependent variable is defined as a dummy that equals 1 when the submitted bid is lower than or equal to $5. The rest of the notation follows the Tobit model. For robustness, we also estimate the same specifications with varying cutoffs to signify low bids, and we obtain similar results. We also ran all of our specifications with alternative cut offs of $6, $7, $7.5 (25^th^ percentile), and $8 with results that parallel those presented in Table 3. Very few bids (n = 11, or 1.43%) were zero bids.

**Table 3:** WTP for wine, tobit model.

	(1)	(2)	(3)	(4)
Organic certification	-3.056***	-3.146***	-4.313***	-4.205***
(more) information	(1.007)	(1.015)	(1.038)	(1.049)
Organic and conventional information (T1)	-2.118**	-1.707*	-2.274***	-2.203***
(all) information	(0.929)	(0.884)	(0.816)	(0.786)
Organic grapes wine	1.650***	1.651***	1.653***	1.654***
	(0.616)	0.616	(0.615)	(0.615)
Organic wine	1.152***	1.152***	1.152***	1.152***
	(0.426)	(0.426)	(0.426)	(0.426)
Organic grape x more info	-0.196	-0.193	-0.197	0.138
	(0.676)	(0.676)	(0.674)	(0.554)
Organic wine x more info	0.138	0.145	0.141	0.138
	(0.554)	(0.555)	(0.554)	(0.553)
Organic grape x all info	0.226	0.224	0.222	0.224
	(0.683)	(0.683)	(0.682)	(0.683)
Organic wine x all info	1.865***	1.865***	1.868***	1.873***
	(0.675)	(0.675)	(0.676)	0.679
Red wine fixed effects	yes	yes	yes	yes
Clustered errors by subject	yes	yes	yes	yes
Socio-economic controls included	no	yes	yes	yes
Wine consumption controls included	no	no	yes	yes
Organic shopping habits	no	no	no	yes
Observations	768	768	768	768
Participants	128	128	128	128
Log Likelihood	-2368.37	-2343.27	-2308.45	-2295.55

Note: Clustered standard errors in parentheses. Baseline: conventional wine, control group.

*** p≤0.01

** p≤0.05

* p≤0.1. Socio-demographics include age, gender, education and income.

While our Tobit specifications capture changes in the *magnitude* of WTP across treatments, the Probit model allows us to examine the impact of information on the *probability of refusal* to buy auctioned wines even at very low prices, which provides additional useful insights about purchasing behavior. Both Tobit and Probit models include a red wine dummy variable to control for often-differing preferences for white and red wines, which allows us to estimate standardized treatment, label, and interaction effects across both red and white wines. All robust standard errors were clustered at the individual participant level.

## Estimation results

The results from the various specifications of the Tobit model are presented in Table 3. The specifications vary based on whether socio-economic controls, information about wine consumption and organic consumption as well as shopping preferences are included or not. While the Tobit model is not linear with respect to underlying consumer preferences (${WTP}_{ijt}^{*}$), the model is linear with respect to measured WTP, which makes the interpretation of the interaction effects more straightforward.

Both organic wines and wines made with organic grapes carry significant WTP premiums, with the premium for wines made with organic grapes slightly higher, at around $1.65, compared with the $1.15 premium for organic wines. Thus, there is no evidence that average WTP is affected by an organic wine stigma in our sample. Rather, subjects are willing to pay a premium of between 10.9% and 15.6% for organic wine and wine made with organic grapes, compared with the WTP for conventional wines.

Confirming the trends we observe in raw cumulative demand for wines, the Tobit estimation results suggest that both information treatments significantly reduce consumer WTP, with an impact of $3.00 to $4.30 in the organic information treatment, and a somewhat smaller reduction of $1.70 to $2.20 in the full information treatment. This is in line with our expectations, outlined in H3 and in previous research [45, 46, 48].

The only significant interaction involves the impact of the full information treatment on demand for organic wine, suggesting that WTP for organic wines is higher when information about both conventional and organic production practices is provided, compared with the same treatment’s impact on conventional wine and wine made with organic grapes. We estimate this increase for WTP for organic wines in the full information treatment to be around $1.87, which suggests that WTP rebounds to levels comparable with the no information (control) treatment. In other words, WTP for organic wines in the full information treatment is estimated at ($1.87-$2.12) = -$0.25, or just 25 cents lower than WTP for organic wines when no information is provided. This is in line with our earlier test suggesting bids for organic wines were not statistically different in the control and full information treatment.

The results for the Probit model specifications are provided in Table 4, with the specifications paralleling those obtained in the Tobit estimation. The Probit results present a slightly different look at consumer behavior. Specifically, we find that wine made with organic grapes is less likely to receive a low bid in both information treatments in the majority of specifications. On the other hand, conventional and organic wines do not differ significantly with respect to the probability of drawing low bids in any of the treatments. Both information treatments increase the probability of submitting low bids compared with the control, significantly for the organic information treatment and mostly significantly for the full information treatment. This suggests that more participants might be avoiding any purchases in both information treatments.

**Table 4:** Bids less than or equal to $5 probability, probit model.

	(1)	(2)	(3)	(4)
Organic certification	0.689**	0.658**	1.112***	1.294***
(more) information	(0.299)	(0.304)	(0.307)	(0.337)
Organic and conventional information (T1)	0.472	0.464	0.712**	0.724**
(all) information	(0.292)	(0.311)	(0.295)	(0.308)
Organic grapes wine	0.073	0.086	0.099	0.084
(0.166)	(0.172)	(0.211)	(0.219)
Organic wine	-0.081	-0.074	-0.126	-0.145
(0.182)	(0.184)	(0.220)	(0.229)
Organic grape x more info	-0.514**	-0.545**	-0.584**	-0.591**
(0.228)	(0.235)	(0.271)	(0.273)
Organic wine x more info	-0.141	-0.153	-0.126	-0.113
(0.222)	(0.224)	(0.256)	(0.263)
Organic grape x all info	-0.408	-0.437*	-0.518*	-0.530*
(0.223)	(0.224)	(0.271)	(0.286)
Organic wine x all info	-0.447	-0.469	-0.468	-0473
(0.292)	(0.293)	(0.333)	(0.352)
Red wine fixed effects	yes	yes	yes	yes
Clustered errors by subject	yes	yes	yes	yes
Socioeconomic controls included	no	yes	yes	yes
Wine consumption controls included	no	no	yes	yes
Organic shopping habits	no	no	no	yes
Observations	768	768	768	768
Participants	128	128	128	128
Log Likelihood	-283.02	-277.53	-250.93	-239.39

Note: Clustered standard errors in parentheses. Baseline: conventional wine, control group.

*** p≤0.01

** p≤0.05

* p≤0.1. Socio-demographics include age, gender, education and income.

## Discussion

### We now discuss the empirical results in light of our hypotheses

*H1*: *Consumer WTP for conventional wine differs from consumer WTP for wine carrying organic labels*.

The evidence for significant WTP premiums for organic wine and wine made with organic grapes strongly supports the first hypothesis. Moreover, our results suggest that our participants do not attach any stigma to organic wines, contrary to some findings reported in the literature. The Probit model results also suggest that some might be more likely to consider wine made with organic grapes when more information is available. This might be driven by the “compromise option” phenomenon, often observed when one of the options available is perceived by consumers to be a middle-ground option [54, 55]; both of the information treatments in the experiment explicitly detail the production standards for wine made with organic grapes, positioning it as a middle option between conventional and organic wines.

*H2*: *Consumer preferences for products with similar but distinct absence labels depend on consumer knowledge about the presence of absence-label ingredients in conventional production practices*.
*H2*.*A As information about conventional winemaking practices is provided*, *the relative valuations of distinct organic labeling standards will change*.

We find no significant change in WTP for wine made with organic grapes relative to WTP for organic wine when we provide information about only the certification standards requirements (p = 0.6383, based on the last Tobit specification). However, once we also add information about conventional winemaking practices, consumers are willing, on average, to pay higher premiums for organic wine than for wine made with organic grape (p = 0.0076, based on the last Tobit specification). This finding is in line with our intuition about absence labels, where information about potential “absentee” ingredients might drive consumer demand for absence certification. While consumer WTP for organic wine is significantly higher than WTP for wine made from organic grapes in the full information treatment, we find that in all other cases wines made with organic grapes carry a small but insignificant premium of around $0.70 (p = 0.1712, based on the last Tobit specification). This suggests that when participants might be unclear about the differences between organic wines and wines made from organic grapes and know little about conventional wine production, they might slightly prefer wine made from organic grapes as a simpler certification standard, but otherwise consider the two labels to be quite similar.

*H3*. *Providing information about production practices will generally decrease WTP for wine*.

Similar to Li et al. [45], we find that any information about production practices and standards requirements significantly reduces consumer WTP for all wines in the experiment (except for organic wine in the full information treatment, discussed above). Similarly, both information treatments increased the probability that participants would make very low bids. We hypothesize that several factors are involved in this result. First, by introducing new and most likely unexpected information, we might have made it harder for participants to align the wines with their preferences, and thus introduced additional cost to the decision-making process. For example, consumers who were previously unaware of the use of egg whites in wine production might be hard pressed to declare whether they prefer the use of organic or conventional egg whites in their wine. In other words, this factor might increase consumer cognitive load, which might explain the reduction in WTP. Second, by introducing information about agricultural production practices, we might have unwittingly diminished the appeal of wine as a highly traditional and romantic product, similar to the aesthetic concepts in wine consumption [47] by making wine more mundane. While our experiment does not enable us to differentiate specific drivers of reduced WTP, both of these findings present an opportunity for future research.

## Conclusion

This paper uses an artefactual experiment to examine consumer WTP for wines under varying certification standards (conventional wine, wine made with organic grapes, and organic wine) when information about certification standards and conventional wine-making practices is available. More generally, this research provides insights into how absence labels shape consumer demand for labeled and conventional products in the presence or absence of information about conventional production practices. In particular, using our sample, we find that the impact of information about certification standards on consumer WTP changes when information about conventional winemaking practices is available: organic wine becomes sharply differentiated from wines made with organic grapes, and exhibits a higher WTP premium.

This paper presents, to the best of our knowledge, the first evidence pertaining to the comparative effects of the two U.S. organic certification standards on consumer WTP for wine. We find that both organic standards confer a WTP premium between 10% and 16% when compared with conventional wine with no evidence of organic wine stigma. Participants’ average WTP is estimated to be similar for wine made with organic grapes and organic wine in the absence of information about conventional winemaking practices. When this information was provided, WTP for organic wines is estimated to be approximately $1.30 higher than that for wine made with organic grapes.

Our findings provide some food for thought for wine producers who are considering adopting a particular certification standard. While wine made with organic grapes carries the highest premium and can be attractive from this perspective, providing additional information reduces consumer WTP in our study. On the other hand, organic wine carries the highest WTP when information about conventional winemaking practices was available. As consumers increasingly pay attention to how their food is produced, this might provide an opening for some winemakers.

Our research also leaves several questions about the motivations and drivers behind the observed participant behavior unanswered, which presents opportunities for future research to expand our understanding of consumer preferences for products with labels that report the absences of certain characteristics. Finally, as our data were generated in a laboratory setting with a convenience sample of wine drinkers, we expect our results to be more indicative of the relative magnitude of the expected effects in the field, rather than of their absolute size [56].

## Supporting information

S1 Dataset Minimal anonymized dataset required for replication. (CSV)
